# Identifying Suitable Supplements to Improve Piglet Survival during Farrowing and Lactation

**DOI:** 10.3390/ani11102912

**Published:** 2021-10-08

**Authors:** Tobias Threadgold, Emma Catharine Greenwood, William Van Wettere

**Affiliations:** School of Animal and Veterinary Sciences, Roseworthy Campus, The University of Adelaide, Mudla Wirra Road, Roseworthy, SA 5371, Australia; tobithreadgold@live.com.au (T.T.); emma.greenwood@adelaide.edu.au (E.C.G.)

**Keywords:** nutrition, pig, piglet viability, sow, supplement

## Abstract

**Simple Summary:**

The death of piglets during birth and prior to weaning is a concern for welfare and affects the profitability of production systems. Four distinct parameters can be identified as having a direct impact on the survival of piglets to weaning. These parameters are stillbirth rate, birth weight and weight variation, daily gain and weaning weight, and colostrum and milk quality. These four parameters were found to benefit from different supplements provided to the sow around the time of farrowing (giving birth). For example, stillbirth could be reduced by supplements in late pregnancy, including forms of arginine, alpha-tocopherol-selenium, uridine, and *Saccharomyces cerevisiae* yeast culture, whereas average daily weight gain and weaning weight of the piglets were closely related to supplements which improved colostrum and milk quality. Therefore, an effective supplement plan to improve piglet survival must consider the circumstances of the individual system and target one or more of the highlighted parameters. It is unlikely that any one supplement will successfully target all four parameters discussed.

**Abstract:**

Piglet mortality during parturition and prior to weaning is an ongoing economic and welfare issue. This review collates the current literature describing the effects of specific dietary supplements on key parameters affecting piglet survival. Four distinct parameters were identified as having a direct impact on the survival of piglets to weaning: stillbirth rate, birth weight and weight variation, daily gain and weaning weight, and colostrum and milk quality. In the primary stage, relevant literature from the past 5 years was reviewed, followed by a secondary review of literature older than 5 years. The focal parameters benefitted from different supplements. For example, stillbirth may be reduced by supplements in late gestation, including forms of arginine, alpha-tocopherol-selenium, uridine, and *Saccharomyces cerevisiae* yeast culture, whereas average daily gain and weaning weight were related closely to supplements which improved colostrum and milk quality, most commonly fats and fatty acids in the form of n-3 polyunsaturated fatty acids, soybean oil, and fish oil, and polysaccharides, such as ginseng polysaccharide. Therefore, an effective supplement plan for piglet mortality reduction must consider the circumstances of the individual system and target one or more of the highlighted parameters.

## 1. Introduction

Piglet mortality during parturition and prior to weaning is an ongoing economic and welfare issue for pig producers in Australia and around the world [1]. Modern breeding sows have been selected for larger litter sizes [2]. However, the benefits of larger litters can be offset by foetal crowding and extended parturition length, which increase the risk of hypoxia, intrapartum death (stillbirth), low birth weight, high within-litter birth weight variation, and more low-viability piglets [2]. As a result, increases in the total number of piglets born does not always result in an equal increase in the number of piglets born alive or number to survive to weaning [3]. Management strategies are required to counteract the negative effects of large litter sizes on the potential of piglets to survive to weaning. Maternal nutrition directly impacts foetal development and nutritional supplementation during late gestation may prove to be an easy-to-adopt strategy to improve piglet outcomes [4].

In recent years, several studies have investigated the potential of specific nutritional supplements to impact foetal growth and development as well as piglet viability. The impacts of these supplements on piglet characteristics associated with survival have been investigated, including piglet birth weight, within-litter variation in piglet birth weight, umbilical blood flow, colostrum quality, transfer of maternal antibodies, farrowing duration, and piglet vigour. All of which are closely related to and, largely, determine piglet viability and pre-weaning mortality [5]. The objective of this review is to summarise the outcomes of these studies and identify which nutritional supplements have the potential to increase piglet survival to weaning. This review differs from those which have come before in its structure, separating current studies into characteristics associated with piglet survival and discussing nutritional supplements within these characteristics. The four main piglet success parameters which this review focuses on are (1) stillbirth rate, (2) birth weight and within-litter variation, (3) average daily gain and weaning weight, and (4) colostrum and milk quality. These characteristics were selected on the basis that they are commonly measured throughout piglet nutrition trial reports and can be objectively compared between trials, as well as all playing a major part in piglet viability and survival. Separation of the review into these sections will allow the review to be used as a resource to more easily isolate individual on-farm piglet viability problems.

## 2. Materials and Methods

This review was conducted initially as part of course requirements for studies undertaken in a Doctor of Veterinary Medicine at the University of Adelaide. The PubMed search engine was used for primary research with the terms “(sow OR gilt OR pig) AND (supplement) AND (offspring or piglet) AND (mortality OR vitality OR survival)”, results from which were combined with an altered set of terms, “(sow OR gilt OR pig) AND (supplement) AND (late gestation)”. A filter was applied to show only articles published in the past 5 years. From that list the author selected only articles which were relevant to the review topic, and finally a list of articles was decided upon. Secondary research of literature older than 5 years was conducted where required to explain or substantiate primary findings. A summary table (Table S1) of studies discussed in this paper has also been made available as Supplementary Information.

## 3. Results

### 3.1. Parameter One: Stillbirth Rate

The number of piglets born alive considers the total litter size minus any stillbirths. In the past there has been a push for larger litter sizes to increase the number of piglets weaned and ultimately sold; however, there is a correlation between larger litter sizes and decreased viability of piglets as well as decreased welfare outcomes [2]. Therefore, in order to increase number of piglets born alive and ultimately the number weaned, production targets may be more easily met by focusing on decreasing stillbirths rather than increasing overall litter size. A major cause of stillbirths and early death of live-born piglets is asphyxia during birth. Therefore, supplements which improve piglet blood oxygen concentration prior to and after birth are especially valuable [6].

#### 3.1.1. Caffeine

The effects of late-gestation caffeine supplementation to the sow on piglets during and immediately after birth have begun to be noticed in recent years. Caffeine has a chemical structure which allows it to cross the placenta and act directly on piglets in utero, where it is documented to have positive effects on the thermoregulatory capacity of piglets as well as provide protection from hypoxia, a major cause of death in piglets born late in parturition [7,8,9]. In a trial by Dearlove et al. (2018), supplementing sow feed with 6 g of caffeine (CAF) per day from day 112 of gestation increased gestation length (CON: 115.6 ± 0.26, CAF: 116.6 ± 0.25, and *p* < 0.05), decreased the total percentage of stillborn piglets (CON: 6.2%, CAF: 2.6%, and *p* < 0.05), and decreased the percentage of sows giving birth to stillborn piglets (CON: 43.3%, CAF: 20.6%, and *p* < 0.05). Oral caffeine supplementation was also shown to be effective at reducing the stillbirth rate per sow by Superchi et al. (2016) at a dose of 27 mg/kg on day 113 of gestation when compared with the group that did not receive the supplement (CON: 2.44, CAF: 0.67, and *p* < 0.05)., although this trial was conducted with only 20 sows [10,11]. A more recent study also supports these positive findings, with the caffeine being given via subcutaneous injection to the vulval area on days 113 and 114 of gestation (with 210 mg/day). Despite the different administration route, this trial also resulted in fewer stillbirths with caffeine administration [12]. A 2017 article by van Wettere et al. found evidence that caffeine, when given in combination with progesterone at the end of gestation, has negative outcomes on piglet health and increased piglet mortality, so this combination should be avoided. The authors stated the mechanism responsible for this negative effect cannot be established from current data, but suggested it may be due to a combined inhibitory effect of caffeine and progesterone on uterine contractility, thereby increasing parturition length [13]. In conclusion, caffeine supplementation to the sow in the days immediately prior to parturition may be a good option to reduce the number of stillborn piglets, especially those caused by intrapartum hypoxia; further repeated trials are needed before any solid conclusions can be reached. More research should be conducted to determine whether oral administration or vulval injection is the most effective route.

#### 3.1.2. Arginine

To date, the most explored supplement in the field of late-gestational sow feeding for piglet health would appear to be arginine, most commonly as L-arginine, but also as L-arginine HCL. Arginine is a precursor to important metabolites such as nitric oxide and polyamines, which are important for angiogenesis (creation of new blood vessels) and vasodilation (widening of blood vessels) [14]. By these mechanisms, arginine enhances the growth of the placenta and improves nutrient and oxygen delivery to the foetus [15]. There are some promising papers on the effects of arginine on piglet survival. A paper by Nuntapaitoon et al. (2018) added 0.5% L-arginine HCL to sow feed from day 85 of gestation until farrowing. They report a 9.8% increase in live-born piglets per litter and an 8.3% decrease in stillborn piglets per litter when compared with the control group, with the authors concluding that this reflects an improvement in angiogenesis and vasodilation [16]. In contrast, evidence that arginine has no effect on the stillbirth rate appears to be more numerous. Articles have tested various concentrations and timings, including supplementation of 1.0% L-arginine into feed from days 85 to 115 of gestation, 1% L-arginine supplementation in late pregnancy, and one testing 1% L-arginine supplementation from multiple different times from day 15 until farrowing and up to day 85 until farrowing have all found no positive effects on the stillbirth rate [17,18,19]. The authors hypothesise that the differences between the reported papers could stem from the nature of the arginine compound including HCL, or perhaps the varying composition of the base diet between trials, which could make a difference to the effect on the stillbirth rate. Further research is required to substantiate the findings of Nuntapaitoon et al. (2018), but given the current evidence arginine supplementation should not be considered a reliable method of stillbirth reduction.

#### 3.1.3. Alpha-Tocopherol-Selenium and Ascorbic Acid

The effect of alpha-tocopherol-selenium on the stillbirth rate in pigs has not been well-researched to date, with only one apparent report. However, based on this single trial this is a supplement which may prove promising and should be researched further to determine its reliability. The older literature that exists on alpha-tocopherol-selenium as a supplement focuses on its effects on maternal colostrum quality and immunoglobulin production rather than on stillbirths, and therefore little is currently understood about its effects in regard to reducing stillbirths [20,21]. The inclusion of ascorbic acid was seemingly not only for its own positive effects as an antioxidant, limiting free radical damage to cells, but also to aid in protecting alpha-tocopherol from oxidative damage (which might reduce the positive effects of its administration) [22,23]. When focusing on reducing stillbirths, supplementation with 1 mL/30 kg BW of alpha-tocopherol-selenium (ATS) (via injection on day 15, 2, and 7, relative to farrowing) in conjunction with 2 g/day/sow of ascorbic acid (AA) in feed (for the last 15 days of pregnancy) significantly increased the average number of live piglets born per sow when compared to the control (CON: 6.666 ± 0.714, ATS + AA: 8.500 ± 0.763, and *p* < 0.05) [23]. Thus, there is very early evidence that alpha-tocopherol-selenium is a likely supplement for reducing the stillbirth rate, but further trials must be conducted to verify the validity of its benefit.

#### 3.1.4. Uridine

Nucleotides are important in numerous biological processes such as growth, immunity, and fatty acid as well as amino acid composition in animals [24,25,26]. Uridine is important for regulating energy homeostasis and protein as well as lipid metabolism, and plays a crucial role in growth performance [27]. Only one article was found on the effect of uridine on the stillbirth rate of piglets, stating that supplementation with 200 g/t of uridine (UR) from day 85 of gestation until farrowing significantly decreased the stillbirth rate when compared to the control group (CON: 7.72% ± 1.861, UR: 3.36 ± 1.141, and *p* < 0.05) [28]. Following these results, the authors hypothesised that uridine could be a crucial nucleotide for foetal development, which could be the cause of the decrease in mortality [28]. For this reason, uridine is another supplement with early, promising results around reducing stillbirths, but further trials must be conducted to verify the validity of its benefit.

#### 3.1.5. *Saccharomyces cerevisiae* (Yeast Culture)

The positive effects of *Saccharomyces cerevisiae* on piglet gut health are well-documented [29,30]. However, only one article exists exploring its effects on the stillbirth rate, the results of which were inconclusive. Supplementation with 4% *Saccharomyces cerevisiae* in the form of refermented-sorghum-dried distiller’s grains with solubles (4% SSDDGS) (a yeast-culture-rich by-product of the ethanol industry), from day 85 of gestation until weaning significantly reduced the litter stillbirth rate when compared to the control group (CON: 10.21% ± 3.22, 4% SSDDGS: 1.16 ± 3.22, and *p* < 0.05) [31]. However, this effect was not observed in a subsequent repeat of the experiment as part of the same trial (CON: 5.16% ± 1.53, 4% SSDDGS: 3.77 ± 1.53, and *p* = 0.369) [31]. The lack of reproduction of the same result across both experiments combined with a lack of other research to provide support indicates that further research is required to decide if supplementation with yeast culture, such as *Saccharomyces cerevisiae,* is a viable option for decreasing the stillbirth rate in pigs.

### 3.2. Parameter Two: Birth Weight and Within-Litter Weight Variation

Following birth, if piglets survive the farrowing process and are not negatively affected by this high-risk period, then their next hurdle is survival post-farrowing. After birth, piglets face thermal challenges and competition with litter mates for colostrum and milk, and high piglet birth weight is important to reduce piglet mortality during this period. During the suckling period, increased piglet birth weight is correlated with increased vitality and colostrum consumption, as well as an increased ability to thermoregulate. Piglets born weighing less than 1 kg can be considered “light for age” and have a decreased pre-weaning survival rate when compared to “non-light for age” piglets [32]. Birth-weight-related impacts on colostrum intake are closely related to piglet survival, with increased colostrum intake per kg of bodyweight positively affecting piglet survival, and an increased time between birth and first suckle having a negative impact [33]. Early colostrum intake provides passive immunity transfer from the sow, and is important for the early development of the piglet immune system [34]. Therefore, as well as increasing piglet birth weight, attempts to decrease within-litter variation is also crucial, as a higher variation leads to unbalanced competition for resources [16].

Foetal growth is primarily determined by nutrient availability, especially amino acids, which are crucial for foetal growth and development [35]. Improper nutrient availability during gestation can also lead to intrauterine-growth-restricted piglets, which have a high instance of intestinal dysfunction [36]. Increasing piglet birth weight and reducing instances of “light for age” piglets is an important factor in reducing overall pre-weaning mortality rates in a production system. As such, the supplements mentioned below all share a common theme of being either amino acids or otherwise providing energy-rich nutrients to the sow for use in foetal development.

#### 3.2.1. Arginine

As in the “stillbirth rate” section above, arginine has also been one of the main supplements to receive research focus for its positive effects on piglet birth weight. As mentioned earlier in this review, arginine is a precursor to important metabolites such as nitric oxide and polyamines, which are important for angiogenesis and vasodilation. By this mechanism, arginine enhances the growth of the placenta and improves nutrient and oxygen delivery to the foetus, which in turn will ultimately allow the foetus increased access to receive everything necessary for growth [15]. A recent study has reported increased the average piglet birth weight and an increase in piglets weighing above 1.35 kg following supplementation with 0.5% L-arginine HCL (ARG) (CON: 59.7%, ARG−0.5: 67.1, and *p* < 0.05), as well as increased piglet blood oxygen concentration at birth (CON: 91%, ARG-0.5: 88%, and *p* < 0.05) [16]. Arginine supplementation (1%) from day 85 to 110 of gestation reduced the number of piglets weighing less than 800 g (CON: 7.48, ARG-1: 1.88, and *p* < 0.05) and increased uniformity in piglet weight at birth without increasing the average birth weight [19]. This finding is consistent with earlier studies, which found that dietary arginine supplementation can benefit embryo survival and foetal development in gilts and sows through improved placental growth, angiogenesis, and oxygen delivery [37]. In contrast, there have also been trials which have found that 1% L-arginine supplementation in late pregnancy did not improve the average birth weight or birth weight distribution [17,18]. Due to the comprehensive evidence supporting the beneficial effects of late-gestation arginine supplementation on piglet birth weight it is still a strong option, despite the trials in which no effect on the stillbirth rate was seen. However, due to the variation in trial results, it may be the case that arginine supplementation will not be effective on all production systems. The authors hypothesise that this may be due to factors such as differences in basal diet or other undocumented circumstances, such as differences in sow genetics and prolificacy, which may in turn create varied results in the success of the supplement on individual piglets.

#### 3.2.2. N-Carbamylglutamate

As discussed in Section 3.1.2 and Section 3.2.1 above, arginine metabolites are essential in placental angiogenesis and growth. This paper has discussed direct supplementation of different forms of arginine to improve piglet health outcomes, but another approach may be to improve intestinal arginine synthesis by supplementation of N-carbamylglutamate (NCG). NCG is not toxic to animals and is known to play an important role in activating the endogenous intestinal synthesis of arginine [38]. A 2004 article by Wu et al. demonstrated elevated plasma arginine levels in piglets at day four of age that had been administered oral NCG at a rate of 50 mg/kg bw every 12 h from birth. The same piglets that received the NCG supplement also demonstrated elevated bodyweight at day four [38]. A more recent study by Wu et al. (2012) investigated the effect of maternal supplementation of 1.0% Arg or 0.1% NCG on sow reproductive performance and found that both treatment categories improved the average birth weight of all piglets born alive compared to the control (CON: 1.46 kg, Arg: 1.62 kg, NCG: 1.59 kg, SEM = 0.02, and *p* < 0.05) [39]. Most recently, Zhang et al. (2014) investigated the effect of maternal NCG supplementation on gilt reproductive performance and found that the live-litter weight was increased by 12.13–19.17% (*p* < 0.05) in groups supplemented with 0.05%, 0.10%, and 0.15% NCG throughout gestation when compared to the control group [40].

#### 3.2.3. 1,3-Butanediol

1,3-Butanediol is a synthetic carbohydrate which is metabolised to beta-hydroxybutyrate, a ketone body [41]. Ketone bodies have been shown, when present in an elevated concentration, to be metabolised preferentially over other fuels. The preferential metabolism of supplemented ketone bodies improves energy-sparing potential in the sow, allowing more energy to be used for gestational growth and development of the piglet, as well as for maternal growth in gilts [41,42]. Supplementation with 4% 1,3-butanediol (1,3-BUT), in place of wheat in the gestation diet from day 90 of gestation until farrowing significantly reduced the percentage of “light for age” (less than 1 kg) progeny at birth (CON: 18.2%, 1,3-BUT: 13.5, and *p* < 0.05), particularly in gilt progeny (CON: 20.9%, 1,3-BUT: 10.8%, and *p* < 0.05). Piglets born to sows from the treatment group also tended (*p* = 0.085) to be heavier (50 g) than the control group [41]. In contrast, late-gestation supplementation of 4.55% 1,3-butanediol had no effect on piglet birth weight [43]. These contrasting results may be due to geographical differences between the trials (Australia and USA) and associated basal diet, climate, and/or breed differences. Due to the low number of contrasting studies which exist around the effects of 1,3-butanediol on piglet birth weight it cannot be recommended at this time; however, future trials conducted with consistent conditions in location, pig breed, and basal diet may provide more answers.

#### 3.2.4. Amino Acids: Glutamine, Serine, and Lysine

Research into the effects of the amino acids glutamine, serine, and lysine on piglet birth weight is limited; however, existing knowledge on the effects of each on the body indicate they are good candidates for exploration. Glutamine is a vital amino acid for energy supply and protein biosynthesis, and plays a signalling role in intestinal gene expression, cell proliferation, and immune function, all of which contribute to the general health, appetite, and therefore daily gain of piglets [36,44]. Serine contributes to multiple important biosynthetic processes including nucleotide synthesis, protein synthesis, glutathione synthesis, phospholipid and sphingolipid metabolism, and the production of precursors which influence one-carbon metabolism [45]. Due to these important roles in biosynthesis, serine is important for cell proliferation and differentiation; therefore, increasing the availability of serine is valuable for foetal development, and could contribute to increased piglet weight at birth. To date, however, only one trial has investigated the effect of glutamine supplementation on piglet birth weight. In this study, supplementation with 1% glutamine (Gln) from day 85 of gestation until farrowing increased average piglet birth weight (CON: 1.34 ± 0.03 kg, Gln: 1.39 ± 0.02, and *p* < 0.05), and decreased birth weight variation from 17.97% to 14.67% [36]. As with glutamine, only one trial was found that tested the effect of serine supplementation on piglet birth weight. Supplementation with 25% serine (Ser) during late gestation increased the average litter weight (CON: 17.82 kg ± 0.30, Ser: 19.41 ± 0.41, and *p* < 0.05) as well as the individual piglet birth weight (CON: 1.47 kg ± 0.02, Ser: 1.69 ± 0.02, and *p* < 0.05) [46]. Sows are likely to be in a negative lysine balance in the last few days before parturition, due to rapid consumption for foetal growth and colostrum production [47,48]. Therefore, maternal supplementation of lysine (Lys) during this period is likely to have a positive outcome. In 2020, Gourley et al. reported an increase in the average piglet bodyweight (CON: 1.289 ± 0.016 kg, Lys: 1.362 ± 0.016, and *p* < 0.05) when gilts received more lysine and energy from day 113 of gestation. While the same effect on piglet bodyweight was not seen in sows the author did report a decrease in overall pre-weaning mortality in treatment group sows (CON: 8.1% ± 0.79, Lys: 5.4 ± 0.79, and *p* < 0.05) [49]. One article alone for each of these supplements is clearly not enough to draw conclusions from, or to recommend glutamine, serine, or increased lysine as ideal supplements for decreasing the piglet stillbirth rate. However, this very early research shows promise for all three amino acids as potential candidates for future research to establish whether they represent a safe and useful additive to sow feed that should be adopted in the pig industry.

### 3.3. Parameter Three: Average Daily Gain and Weaning Weight

Within this review, average daily gain has been found to be closely related to the quality of milk and colostrum. High-quality colostrum and milk contains high concentrations of nutrients required for piglet growth, development, and survival [20]. One major measure of colostrum quality explored in this report is the content of immune molecules such as immunoglobulin G (IgG) [20]. It is therefore likely that supplements which improve colostrum quality contribute to stronger immune health of the piglet, allowing them to waste less energy on fighting pathogens and lose less nutrients through pathogen-induced diarrhoea, ultimately allowing the piglets to put these nutrients into growth. Additionally, supplements which improve colostrum and milk quality improve the quality and quantity of nutrients available to the sucking piglet for body growth and development and, therefore, the following section focuses on supplements to increase the average daily piglet weight gain (ADG). However, through our discussions it will become evident that this is most reliably improved by increases in milk and colostrum quality. Despite this, some supplements which act by other mechanisms do show promise in the early stages of research.

#### 3.3.1. Fatty-Acid-Rich Supplements

The positive effect of the supplementation of soybean oil or fish oil on litter weight gain can be explained by their positive impact on milk fat composition and colostrum antibody content, as also later explored further in the milk and colostrum quality section below. One trial by Jin et al. (2017) found that supplementation with 2.9% soybean oil (SO) or 3.0% fish oil (FO) from day 90 of gestation until farrowing followed by supplementation with 3.8% soybean oil or 3.9% fish oil throughout farrowing resulted in a significant increase in the overall litter weight gain up to weaning when compared to the control group (CON: 27.69 kg ± 1.53, SO: 33.41 kg ± 1.53, FO:36.73 kg ± 1.53, and *p* < 0.01) [50]. Another trial by Chen et al. (2019) found that supplementation with 68.2 g/kg n-3 polyunsaturated fatty acids in late gestation and lactation tended to increase the piglet bodyweight gain (CON: 186 g/d ± 8.27, n-3 PUFA:204 g/d ± 8.27, and *p* = 0.302) [51]. Finally, a trial by Hasan et al. (2018) found that supplementation with 5 g/day of a composition of 8% resin acid and 90% free fatty acids (RAC) from one week before expected farrowing until farrowing enhanced piglet weight at 3 to 4 weeks in two out of three herds (herd one: CON-1: 7.079 kg ± 159.7, RAC-1: 6.770 kg ± 139.6. Herd two: CON-2: 6.562 kg ± 168.8, RAC-2: 6.939 kg ± 130.7, and *p* = 0.04. Herd three: CON-3: 7.785 kg ± 108.1, RAC-3: 7.872 kg ± 86.2, and *p* = 0.07) [52]. Interestingly, these were the same two out of three herds which also showed increased colostrum quality, further highlighting the correlation between enhanced colostrum quality and piglet weight gain. One reason for the varied result in one herd out of three could be related to the fact that the study followed one herd in the Netherlands whereas the other two were in Finland, introducing an inconsistency between the trials and highlighting possible genetic or management effects of the response to this supplementation. These trials are a strong indication that fatty-acid-rich supplements have the potential to increase piglet weight gain through positive impacts on milk and or colostrum quality in some, if not all, herds.

#### 3.3.2. *Saccharomyces cerevisiae* (Yeast Culture)

The positive effects of *Saccharomyces cerevisiae* yeast culture on piglet gut health are well-documented. Previously, yeast culture has increased the growth performance of pigs, the reason for which was suggested to be improving villus height, gut immune response, and nutrient digestibility [53]. Therefore, increased gut health and development in piglets may be the mechanism by which *Saccharomyces cerevisiae* increases piglet average daily gain and weaning weight. A trial by Song et al. (2017) showed that supplementation to the sow with 4% *Saccharomyces cerevisiae* refermented-sorghum-dried distiller’s grains with solubles (4% SSDDGS) from day 85 of gestation until weaning significantly increased individual piglet weight at weaning (CON: 7.32 kg ± 0.19, 4% SSDDGS: 7.88 kg ± 0.19, and *p* = 0.025) and tended to increase piglet weight gain (CON: 5.94 kg ± 0.16, 4% SSDDGS: 6.28 ± 0.16, and *p* = 0.079) and litter weaning weight (CON: 65.52 ± 9.11, 4% SSDDGS: 85.63 ± 9.11, and *p* = 0.105) when compared to the control group. Song et al. (2017) suggested that the mechanism behind this positive effect may be attributed to increased milk quality and yield, as well as nutrient digestibility, further highlighting the relationship between milk quality and average daily gain [31]. These results support those of Kim et al. (2018), in which in-feed supplementation with 12 g of yeast culture (YC) per day from day 35 to day 109 of pregnancy, followed by 15 g of yeast culture per day until day 21 of lactation, significantly increased piglet daily weight gain in both primiparous and multiparous groups (primiparous: CON: 245.1 g/d ± 3.0, YC: 267.7 g/d ± 3.0. Multiparous: CON: 227.9 g/d ± 3.0, YC: 241.4 g/d ± 3.0., and *p* = 0.013) and litter weaning weight (primiparous: CON: 70.0 kg ± 0.7, YC: 74.8 kg ± 0.7. Multiparous: CON: 65.7 kg ± 0.7, YC: 69.9 kg ± 0.7, and *p* = 0.004). The mechanisms behind this improvement were related to improvements in sow milk quality and production in addition to improved nutrient digestibility [54]. Since then, two separate articles published by Kiros et al. (2018) and Kiros et al. (2019) have reported a positive effect of *Saccharomyces cerevisiae* supplementation on piglet weight gain; however, these supplements were given directly to piglets post-partum in a solution rather than via supplementation of the sow, and these improvements were attributed to improvements in piglet digestive health and reduction in pathogenic bacteria in the gut, rather than to improvements in milk quality from the sow [29,30]. These trials are a strong indication that *Saccharomyces cerevisiae* supplementation is an effective way to increase piglet gut health and average daily gain, and presents the possibility that positive effects may be seen regardless of whether supplementation is to the sow or directly to the piglets, despite varied mechanisms.

#### 3.3.3. Caffeine

The positive effects of maternal caffeine supplementation on the piglet respiratory system and general piglet vigour were explored earlier in this paper. Specifically, caffeine increased piglet gas exchange capacity and vigour at birth, thereby decreasing the proportion of stillborn piglets. However, it may also affect piglet success post-farrowing, as the increased piglet vigour at birth from the stimulant effects of caffeine allow better access to teats which provide the greatest level of nutrition for daily gain, as well as colostrum in early lactation for the passive transfer of immunity [7,8,12]. The effects of maternal caffeine supplementation on piglet bodyweight gain are inconclusive, with one study by Sánchez-Salcedo et al. (2019) finding that supplementation with 210 mg/day of caffeine via subcutaneous injection to the vulval area on days 113 and 114 of gestation increased bodyweight gain in piglets at day 21 (CON: 6.52 kg ± 0.25, CAF: 6.87 ± 0.18, and *p* < 0.05), but another by Dearlove et al. (2018) finding that oral supplementation with 6 g of caffeine per day from day 112 of gestation had no such effect on piglet bodyweight [11,12]. There are multiple differences in these trials which could explain the inconsistency in results, including the dosage and method of administration; therefore, further research is required in this area to determine under what circumstances this supplementation successfully increases birth weight consistently.

#### 3.3.4. *Enterococcus faecium*

*Enterococcus faecium* is a probiotic known to provide beneficial effects in pigs through the competitive exclusion of pathogenic bacteria, thereby promoting good digestive health and reducing diarrhoea. Probiotics such as *E. faecium* can be transferred from sow to piglet by contact with maternal faeces [55,56]. However, despite these benefits, only one trial was identified exploring the effects of *E. faecium* on piglet daily weight gain. A trial by Lan and Kim (2020) showed that supplementation with 0.025% (E-0.025) or 0.05% (E-0.5) *E. faecium* from 14 days before farrowing until weaning increased piglet weaning weight (CON: 7.54 kg ± 0.07, E-0.025: 7.99 kg ± 0.07, E-0.05: 8.19 kg ± 0.07, and *p* < 0.00) and average daily gain (CON: 233.49 g ± 2.85, E-0.025: 252.60 g ± 2.85, E-0.05: 261.07 ± 2.85, and *p* < 0.00), giving a promising first look at potential advantages in this area [57]. However, as with so many of the previously explored supplements, research into this area is new and minimal; further research is therefore needed to be able to draw any real conclusions regarding the practicality and efficacy of this supplement.

#### 3.3.5. Serine

The important role of serine in biosynthetic processes is discussed in the birth weight and within-litter variation section above. The impact of maternal serine supplementation on average daily gain in piglets can likely be explained by a previous study on mice, which found that the dietary inclusion of L-serine significantly increased L-serine levels in milk. Interestingly, however, the same study found that L-serine supplementation also caused a significant decrease in glutamic acid, tau, L-alanine, and D-alanine, and led to decreased bodyweight of offspring in the L-serine-supplemented group [58]. However, in further studies in pigs these negative effects were not noted [46,58]. Prior to the study mentioned above by He et al. (2020), the effects of maternal serine supplementation during gestation and lactation were unknown. These further studies in pigs found contrasting results to the mice studies, with supplementation of 25% L-serine (L-Ser) during late gestation and lactation significantly increasing the average litter weight (CON: 17.82 kg ± 0.30, L-Ser: 19.41 ± 0.41, and *p* < 0.05) as well as individual piglet weight at day 21 of lactation [46]. Although a single trial conducted in pigs does not provide sufficient evidence to recommend serine as an ideal supplement for increasing piglet birth weight, it does represent a promising candidate for future studies, especially considering the positive results reported in mice.

#### 3.3.6. Dietary Fibre Source

Dietary fibre forms a key component of many pig diets by direct processes, such as gut fill, and indirect processes, including the modulation of how other nutrients and chemicals are absorbed [59]. A variety of fibre sources are used in pig diets, often depending on local availability and economics; however, it is important to note there are differences in nutritive properties of different fibre sources [59,60]. It is therefore reasonable to assume that some fibre sources will be more appropriate than others when considering piglet health and survival. A 2019 article by Shang et al. (2019) explored the effects of different maternal dietary fibre sources on piglet health by comparing supplementation with sugar beet pulp (SBP) compared with wheat bran (WB). Shang et al. reported improvement in average daily gain in the group fed SBP as compared to the control and WB groups (CON: 196 g/d, SBP: 221 g/d, WB: 206 g/d, and *p* = 0.01) [61]. Another 2019 article by Agyekum et al. compared oat hay with wheat hay as maternal dietary fibre sources, and found that the average piglet weight at weaning was marginally higher in the oat hay group compared to the control and to those fed wheat hay (CON: 6.87 kg, oat: 7.02 kg, wheat: 6.86 kg, and *p* < 0.05) [62]. Although there are no significant data to indicate which specific fibre sources should be preferred by any given producer, these results indicate that the choice of maternal fibre source plays an important role in piglet growth.

### 3.4. Parameter Four: Colostrum and Milk Quality

Piglets rely on colostrum intake for the passive transfer of immunity from the sow as, in contrast to other species (including humans), no vertical transfer of immunoglobulins occurs in pigs. It has been demonstrated that there is a clear positive correlation between piglet colostrum intake and average daily gain as well as survival to weaning [33]. As previously mentioned, milk quality determines the quantity and quality of nutrients available to the sucking piglet for body growth and development, and is therefore important for piglet survival throughout the suckling period.

#### 3.4.1. Fatty-Acid-Rich Supplements

Supplements of, or rich in, fatty acids appear to be the most effective at increasing milk and colostrum quality, often leading to an increase in not only milk and colostrum fat content but also immune molecules, with the latter improving the passive transfer of immunity from the sow to their progeny. Fatty-acid-rich supplements and the relationship between milk and colostrum quality with piglet weight gain were already discussed in the piglet weight gain section above, namely that better milk and colostrum quality is positively correlated with increased piglet weight gain. Supplementation with 68.2 g/kg n-3 polyunsaturated fatty acids (n-3 PUFA), 7.75 g/kg medium-chain fatty acids (MCFA), and 1 g/kg sodium butyrate (SB) in late gestation and lactation each lead to significant increases in colostrum fat (CON: 3.67%, SB: 4.09%, MCFA: 4.47%, n-3 PUFA: 5.07%, SEM: 0.2, and *p* < 0.001) and protein content (CON: 12.3%, SB: 16.9%, MCFA: 15.3%, n-3 PUFA: 19.2%, SEM: 0.63, and *p* < 0.001), as well in levels of IgA (CON: 5.80 μg/mL, SB: 18.5 μg/mL, MCFA: 6.70 μg/mL, n-3 PUFA: 6.98 μg/mL, SEM: 0.71, and *p* < 0.001), IgG (CON: 45.1 μg/mL, SB: 169 μg/mL, MCFA: 81.3 μg/mL, n-3 PUFA: 98.0 μg/mL, SEM: 0.2, and *p* < 0.001), and IgM (CON: 45.1 μg/mL, SB: 165 μg/mL, MCFA: 61.2 μg/mL, n-3 PUFA: 56.7 μg/mL, SEM: 0.2, and *p* < 0.001). Of these, n-3 polyunsaturated fatty acids were identified as the most effective treatment to increase the fat and protein content in colostrum [51]. Supplementation with 5 g/day of a supplement consisting of 8% resin acid and 90% free fatty acids from one week before expected farrowing until farrowing enhanced colostrum production, especially colostrum IgG concentration; however, this result was only significant in two out of three test herds (herd one: CON-1: 70.9 mg/mL ± 5.4, RAC-1: 86.3 mg/mL ± 5.27. Herd two: CON-2: 65.5 mg/mL ± 4.2, RAC-2: 76.5 mg/mL ±3.2, and *p* = 0.04. Herd three: CON-3: 92.1 mg/mL ± 6.6, RAC-3: 108.9 mg/mL ± 7.3, and *p* = 0.007). As mentioned above, there was a geographical difference between one of these three trials and the other two, which could explain the difference in the results between herds [52].

Finally, a report by Jin et al. (2017) found that supplementation with 2.9% soybean oil from day 90 of gestation until farrowing followed by supplementation with 3.8% soybean oil throughout farrowing resulted in a significant increase in milk fat content (CON: 5.88% ± 0.18, SO: 7.15% ± 0.18, and *p* < 0.01). The same trial found that supplementation with 3.0% fish oil from day 90 of gestation until farrowing followed by supplementation with 3.9% fish oil throughout farrowing significantly increased milk fat content (CON: 5.88% ± 0.18, FO: 6.56% ± 0.18, and *p* < 0.01) as well as IgG and IgM levels in colostrum (IgG; CON: 48.51 g/L ± 1.86, FO: 72.5 g/L ± 1.88, and *p* < 0.01) (IgM; CON: 3.07 g/L ± 0.17, FO: 4.16 g/L ± 0.17, and *p* < 0.01) and milk (IgG; CON: 1.53 g/L ± 0.08, FO: 1.77 g/L ± 0.08, and *p* < 0.01) (IgM; CON: 1.87 g/L ± 0.10, FO: 2.48 g/L ± 0.10, and *p* < 0.01) [50]. Overall, there is strong evidence to support the use of fatty-acid-rich supplements to improve milk and colostrum quality; however, there may be variation in the degree of the effect depending on the supplement used combined with varied production systems and genetics; there is insufficient research available to declare any source the best.

#### 3.4.2. *Saccharomyces cerevisiae* (Yeast Culture)

The positive effects of maternal *Saccharomyces cerevisiae* yeast culture supplementation on piglet daily growth as discussed above in the average daily gain and weaning weight section have been accredited to improvements in sow milk quality and yield. To reiterate, Song et al. (2017) and Kim et al. (2008) both found benefits of yeast culture supplementation on piglet weight gain and suggested that the mechanism behind this positive effect can be attributed to increased milk quality and yield, as well as nutrient digestibility [31,54].

#### 3.4.3. Arginine

Nitric oxide and polyamines converted from arginine are proposed to support mammary tissue growth, thereby improving lactogenesis (milk production) and nutritional support for sucking piglets [63]. Evidence of this claim was largely unobserved in the literature examined in this review [17,19,64]. However, an improvement in colostrum quality was seen in some cases [16,65]. Specifically, dietary supplementation with 1.0% L-arginine HCL from 85 days of gestation until farrowing increased colostrum IgG at 1 h after the onset of farrowing when compared to the control group (CON: 85 mg/mL, Arg-1.0: 116 mg/mL, and *p* < 0.05) [16]. At this stage, arginine is not a top candidate for milk and colostrum quality improvement; however, there is evidence to suggest that it may be beneficial in that regard in some specific circumstances yet unidentified, such as in sows predisposed to poor mammary tissue growth leading to insufficient lactogenesis or piglet support during lactation.

#### 3.4.4. Other Promising Early Research

The effect of ginseng polysaccharides on sow or gilt milk and colostrum quality are largely unexplored. However, Xi et al. (2017) demonstrated that supplementation with up to 400 mg/kg of ginseng polysaccharides from day 90 of gestation until weaning significantly increased concentrations of antioxidant enzymes and immune molecules in milk and colostrum, including IgG, IL-2, IL-6, and TNF-α (but not IFN-γ) [66]. As with ginseng polysaccharides discussed above, the effect of garcinol supplementation on milk and colostrum quality is also largely unexplored in pigs. However, Wang et al. (2019) reported an increased crude percentage, as well as IgA and IgG levels in colostrum and milk produced by sows receiving 200–600 mg/kg of feed of garcinol from day 90 of gestation until the end of lactation [67]. The mechanism by which either supplement increases milk and colostrum quality is unknown; however, both have an effect on the oxidative status of the sow, increasing antioxidant status. In addition to this, there is evidence to suggest that sow oxidative status affects milk composition [68]. This is not enough evidence to make a recommendation on whether ginseng polysaccharides or garcinol supplementation are viable options for improving milk and colostrum quality in pigs; however, both have shown promising initial signs and should be watched for further research to be performed in order to determine the consistency of positive effects across different dose ranges and on different herds and production systems.

### 3.5. Pre-Weaning Mortality

Of all the articles reviewed in this paper, a significant number did not explicitly report an effect on pre-weaning mortality, or reported that positive effects of the supplement did not ultimately result in a significant reduction in pre-weaning mortality. Reasons for this may lie in shortcomings in other parts of the production system, meaning that the parameters improved in the trials were not the most important factor-limiting issues for piglet survival in those systems. Put more simply, it is hypothesised that the lack of effect on total pre-weaning mortality of the researched supplements may be the dilution of their beneficial effects by the many factors affecting piglet success in the overall production system. This indicates that when seeking to improve pre-weaning mortality in a pig production system it may be important to first identify which parameters of foetal and placental development, birth weight, or post-partum growth and development are most in need of improvement, and targeting those specifically, to see beneficial effects in the ultimate “end point”.

## 4. Conclusions

It was decided to focus this review on four distinct parameters, which were identified as having a direct impact on the survival of piglets to weaning. These parameters were stillbirth rate, birth weight and within-litter birth weight variation, average daily gain and weaning weight, and colostrum and milk quality. For improvement to the stillbirth rate, caffeine supplementation orally or by vulval injection in the days immediately prior to parturition was found to be the most effective method supported by the research to date; however, further research is required before this can be determined with absolute certainty. It is suggested that L-arginine given throughout late gestation is the most promising supplement for improving piglet birth weight. Piglet weight gain was related to milk and colostrum quality, both of which are best improved by the introduction of fatty-acid-rich supplements, with strong evidence indicating that supplementation with yeast culture is also a viable option for improving milk quality and yield. In some cases, significant improvements to pre-weaning mortality were reported; however, it was not uncommon for researchers to report no significant decrease in pre-weaning mortality despite improvements in these parameters, indicating the existence of significant limitations in other parts of the production system, which ultimately dilute the effects of these supplements on the overall production system efficiency. The supplements identified in this review may therefore be an important tool for decreasing piglet mortality; however, no single recommendation can be made to apply to all production systems, and an effective late-gestation and early lactation supplement plan for piglet mortality reduction must consider the circumstances of the individual system and target one more or more limiting issues of piglet gestation, birth weight, or post-partum growth and development. Among the supplements described in this review many newly researched compounds were identified to be promising in the currently early stage, but were still too under-researched to make recommendations on their usage. This is important to note, as there is often only one paper on a single supplement which can be interpreted. We would certainly suggest that further research into these supplements is required to find what place they have, if any, in improving piglet health parameters and decreasing pre-weaning mortality.

## Data Availability

No new data were created or analyzed in this study. Data sharing is not applicable to this article.

## Supplementary Materials

The following are available online at https://www.mdpi.com/article/10.3390/ani11102912/s1, Table S1: Summary table of sources discussed in the main paper.
