# Supplementary material for: Identifying Suitable Supplements to Improve Piglet Survival during Farrowing and Lactation
Source: Animals (Basel). 2021 Oct 8;11(10):2912. doi: 10.3390/ani11102912 (PMC8532790; doi:10.3390/ani11102912)
Supplement: Supplementary file 1 [file animals-11-02912-s001.zip › animals-1334471-supplementary.pdf]

Table S1: Summary table of sources discussed in the main paper.

Source and list number	Supplement	Dose and Timing	Trial effects discussed*				
			Stillbirth	Birth weight	ADG & weaning weight	Colostrum quality	Milk quality
Dearlove et al. 2018 (11)	Caffeine	Sows fed 6g per day from day 112 of gestation until farrowing	+	/	/	/	/
Superchi et al. 2016 (10)	Caffeine	Sows fed 27mg/kg on day 113 of gestation	+	/	/	/	/
Sánchez-Salcedo et al. 2019 (12)	Caffiene	Sows given 210mg via subcutaneous injection on days 113 and 114 of gestation	+	/	+	/	/
van Wettere et al. 2018 (13)	Caffiene and Progesterone	Sows fed 5mL of Regumate Porcine daily from days 111 to 113 of gestation, and 2.4g of caffeine per kg diet from day 113 of gestation until parturition	-	/	/	/	/
Nuntapaitoon et al 2018 (16)	L-Arginine HCL	Sows fed 0.5% or 1.0% L-Arginine HCL from day 85 of gestation until farrowing	+	+	/	+	/
Bass et al. 2017 (17)	L-Arginine	Sows fed 1.0% L-Arginine in late gestation diet	/	/	/	/	/
Hines et al. 2019 (18)	L-Arginine	Sows fed 1.0% L-Arginine in late gestation diet	/	/	/	/	/
Moreira et al. 2020 (19)	L-Arginine	Sows fed 1.0% L-Arginine in late gestation diet	/	+	/	/	/
Krogh et al. 2017 (65)	Crystalline Arginine	Sows fed 25g per day of crystalline Arginine from day 30 of gestation until farrowing	/	/	/	+	/
Gaykwad et al. 2019 (23)	Alpha-tocopherol-selenium and ascorbic acid	Sows administered 1mL/30kg of alpha-tocopherol-selenium via injection on day -15, 2, and 7 relative to farrowing in conjunction with 2g per day per sow of ascorbic acid in feed for the last 15 days of pregnancy	+	/	/	/	/
Gao et al. 2020 (28)	Uridine	Sows fed 200g/t of uridine from day 85 of gestation until farrowing	+	/	/	/	/

Source and list number	Supplement	Dose and Timing	Trial effects discussed*				
			Stillbirth	Birth weight	ADG & weaning weight	Colostrum quality	Milk quality
Song et al. 2017 (31)	Yeast Culture	Sows fed 4% Saccharomyces cerevisiae refermented sorghum dried distiller''s grains with solubles from day 85 of gestation until weaning	~	/	+	/	+
Wu et al. 2012 (39)	N-Carbamylglutamate	Sows fed 0.1% N-Carbamylglutamate throughout gestation	/	+	/	/	/
Zhang et al. 2014 (40)	N-Carbamylglutamate	Sows fed 0.05%, 0.10%, or 0.15% N-Carbamylglutamate throughout gestation	/	+	/	/	/
Wijesiriwardana et al. 2019 (41)	1,3-Butanediol	Sows fed 4% 1,3-Butanediol in place of wheat in the gestation diet from day 90 of gestation until farrowing	/	+	/	/	/
Stahly et al. 2014 (43)	1,3-Butanediol	Sows fed 4.55% 1,3-butanediol in the late gestation diet	/	/	/	/	/
Zhu et al. 2018 (36)	Glutamine	Sows fed 1% glutamine from day 85 of gestation until farrowing	/	+	/	/	/
He et al. 2020 (46)	Serine	Sows fed 25% serine in the late gestation diet	/	+	+	/	/
Gourley et al. 2020 (49)	Lysine	Sows and gilts fed increased levels of lysine and energy from day 113 of gestation until farrowing	/	~	/	/	/
Jin et al. 2017 (50)	Soybean Oil	Sows fed 2.9% soybean oil from day 90 of gestation until farrowing and 3.8% soybean oil throughout farrowing	/	/	+	/	+
Jin et al. 2017 (50)	Fish Oil	Sows fed 3.0% fish oil from day 90 of gestation until farrowing and 3.9% fish oil throughout farrowing	/	/	+	+	+
Daneshmand et al. 2017 (51)	Medium Chain Fatty Acids	Sows fed 7.75g per kg medium-chain fatty acids throughout gestation	/	/	/	+	/
Daneshmand et al. 2017 (51)	Sodium Butyrate	Sows fed 1g/kg sodium butyrate throughout gestation	/	/	/	+	/

Source and list number	Supplement	Dose and Timing	Trial effects discussed*				
			Stillbirth	Birth weight	ADG & weaning weight	Colostrum quality	Milk quality
Daneshmand et al. 2017 (51)	n-3 polyunsaturated fatty acids	Sows fed 68.2g per kg n-3 polyunsaturated fatty acids in late gestation and lactation diets	/	/	+	+	/
Hansen et al. 2018 (52)	Renin Acid Composition	Sows fed 5g per day of a composition of 8% resin acid and 90% free fatty acids from one week before expected farrowing until farrowing	/	/	~	~	/
Lan & Kim 2020 (57)	Enterococcus faecium	*E. faecium* added to sow diet from 14 days before farrowing until weaning	/	/	+	/	/
Shang et al. 2019 (41)	Sugar Beat Pulp as a fibre source	Sows fed sugar beet pulp as daily fibre source	/	/	+	/	/
Agyekum et al. 2019 (59)	Oat Hay as a fibre source	Sows fed oat hay as daily fibre source	/	/	+	/	
Xi et al. 2017 (66)	Ginseng Polysaccharides	Sows fed 400mg per kg of ginseng polysaccharides from day 90 of gestation until weaning	/	/	/	+	+
Wang et al. 2019 (67)	Garcinol	Sows fed 200 – 600 mg per kg of garcinol from day 90 of gestation until the end of lactation	/	/	/	+	+

* Trial effects in the presented papers have been highlighted using the following symbols; Positive result: +, Negative result: -, Mixed result: ~, Neutral or not discussed result: /.
